# Antioxidant Capacity, Total Phenolic Content and Phytochemical Profile of Canned Dandelion (*Taraxacum officinale* L.) Flowers

**DOI:** 10.17113/ftb.63.03.25.8738

**Published:** 2025-09-05

**Authors:** Ayca Gülhan, Mehmet Fuat Gülhan, Oğuz Çakır, Cihan Düşgün, Mustafa Abdullah Yılmaz

**Affiliations:** 1 Department of Food Technology, Vocational School of Technical Sciences, Aksaray University, 68100, Aksaray, Türkiye; 2 Department of Medicinal and Aromatic Plants, Vocational School of Technical Sciences, Aksaray University, 68100, Aksaray, Türkiye; 3 Department of Nutrition and Dietetic, Atatürk Faculty of Health Sciences, Dicle University, Diyarbakır, 21280, Türkiye; 4 Science and Technology Application and Research Center, Dicle University, Diyarbakir, 21280, Türkiye; 5 Department of Biology, Faculty of Science Literature, Niğde Ömer Halisdemir University, 51200, Niğde, Türkiye; 6 Mustafa Abdullah Yılmaz, Department of Analytical Chemistry, Faculty of Pharmacy and Science and Technology Research and Application Center, Dicle University, Diyarbakir, 21280, Türkiye

**Keywords:** dandelion flower, canned food, antioxidant activity, phytochemicals, LC-MS/MS

## Abstract

**Research background:**

Dandelion flowers have a very short shelf life. The canning process is known not only to stabilise food and preserve its nutritional content at a high level, but also to significantly extend its shelf life. For this reason, canned dandelion flowers are believed to be beneficial for both consumers and the gastronomy sector.

**Experimental approach:**

In this study, fresh dandelion (*Taraxacum officinale* L.) flowers were canned using sucrose syrup with different (20 and 30) degrees of Brix (°Bx) as filling medium and stored at 25 °C for 30 days. A total of 56 phytochemicals were identified using liquid chromatography-tandem mass spectrometry (LC-MS/MS), while the *in vitro* antioxidant activity of 2,2-diphenyl-1-picrylhydrazyl (DPPH) and cupric reducing antioxidant capacity (CUPRAC) and the total phenolic content (TPC) were analysed in both the canned flowers and the syrup at different storage times (on days 10, 20 and 30).

**Results and conclusions:**

The antioxidant activities of fresh dandelion flowers were 89.6 % and 0.8 mmol Trolox equivalents (TE) per gram, respectively. The lowest DPPH (41.4 %) and CUPRAC expressed as TE (0.3 mmol/g) activities were observed on day 20 in samples stored in the 30 °Bx syrup. The TPC in fresh flowers, expressed as gallic acid equivalents (GAE) per g of extract, was 367.4 mg/g. The highest TPC in canned flowers was determined on day 10 in the samples in syrup with both °Bx. LC-MS/MS analysis identified 24 phytochemicals in fresh flowers, including quinic acid, luteolin, siranoside, chlorogenic acid, fumaric acid, caffeic acid, protocatechuic acid, quercetin, cosmosiin, isoquercitrin and apigenin. A decrease in the polyphenol content of canned flowers was observed during storage. The results indicate that canning dandelion flowers in a 30 °Bx syrup and storing them for 20 days preserved their phenolic content and antioxidant capacity.

**Novelty and scientific contribution:**

Numerous studies in the literature focus on extending the shelf life of fruit and vegetables by the canning method. However, this study fills a gap in the literature by successfully applying the canning technique to edible flowers for the first time. Furthermore, the results of this study contribute to future research on the potential commercialisation of canned dandelion flowers as a food product.

## INTRODUCTION

Since ancient times, edible flowers have traditionally been consumed as an alternative to medicines or as part of the culinary art. These flowers are highly valued for their ability to enrich dishes with aroma and vibrant colour and are used in different beverages, salads, soups, sauces, cakes, purees, omelettes and desserts. In addition to their aesthetic appeal and pleasant aroma, edible flowers have health-promoting effects and a high nutritional value (*1*, *2*). Researchers have identified edible flowers as innovative natural sources of bioactive compounds (*2*, *3*). Consequently, scientific interest in the nutritional value and phytochemical profiles of edible flowers has steadily grown (*3*, *4*). Phytochemicals such as phenolics and flavonoids have been reported to significantly reduce the risk of health problems, including cardiovascular diseases, obesity and cancer (*4*, *5*). Compared to fruit and vegetables, edible flowers contain higher concentrations of antioxidant compounds, such as vitamin C, carotenoids, anthocyanins and polyphenols (*6*). In Europe, the pharmaceutical use of the edible plant dandelion (*Taraxacum officinale* L.) has a long history in traditional medicine. Dandelion flowers, in particular, are known for their cough-relieving and immune-boosting properties and are traditionally used in Central and Eastern Europe, particularly in countries like Croatia and Poland, to make a syrup called "honey" (*7*). Luteolin and its 7-O-glycoside, which are abundant in dandelion flowers, inhibit the production of nitric oxide and prostaglandin E2 in macrophages stimulated by bacterial lipopolysaccharides. Furthermore, extracts from dandelion flowers have been shown to inhibit liposome oxidation *in vitro* and protect against DNA damage caused by free peroxide (O_2_˙‾) and hydroxyl (OH˙) radicals (*8*). *In vivo* studies have shown that dandelion flower extracts have a higher flavonoid content than other plant organs, which contributes to stronger antioxidant properties (*9*, *10*). Processed edible flower products offer several advantages over fresh flowers. Processed products are safer to consume, as the high water content of fresh flowers can lead to rapid proliferation of microorganisms (*11*). Preservation techniques, such as processing, can extend the shelf life of edible flowers while maintaining their sensory properties over longer periods of time. Canning is a preservation method in which products are packed in hermetically sealed and sterilised containers, which preserves product quality for a long time (*12*). Combining a specific temperature and time, the canning process eliminates food pathogens and inactivates enzymes responsible for quality deterioration during storage. As a result, the final products are stable at ambient temperature and have a long shelf life (*13*). In the food industry, many types of fruit and vegetables are preserved in cans or glass jars with suitable sucrose syrups or brines (*14*). Studies have shown that canned fruit has a nutritional value comparable to that of fresh fruit (*15*, *16*). However, concerns persist about the potential reduction of bioactive compounds in foods due to the type of processing and extraction conditions (*17*). Ultrasonic technology is increasingly used in food processing, preservation and extraction. This method offers energy efficiency, effective extraction and protection for heat-sensitive compounds through the use of low temperatures (*18*, *19*). Edible flowers have a short shelf life and limited production time, which necessitates preservation technologies. Nevertheless, many preservation techniques for edible flowers have not yet been sufficiently explored (*20*). In this study, the canning technique was applied to fresh dandelion flowers for the first time. The flowers were canned using sucrose syrups with different degrees of Brix (20 and 30) as a filling medium and stored at 25 °C for 30 days. The antioxidant activity, TPC, and 56 phytochemicals identified by LC-MS/MS were analysed in samples collected on days 10, 20 and 30 of storage. The results showed the transfer of bioactive compounds from fresh dandelion flowers to the syrup and the quantitative changes in the flowers during storage.

## MATERIALS AND METHODS

### Plant material

The dandelion (*Taraxacum officinale* L.) samples selected for this study were harvested from the traffic-free area of Yeşilova Village in Aksaray Province, Türkiye (38°24'37.7"N, 33°51'18.7"E) in September 2023. Fresh samples were prepared for analysis on the same day. The plant species was identified by Prof. Dr. Mehmet Fuat Gülhan from the Department of Medicinal and Aromatic Plants at Aksaray University, Türkiye.

### Preparation of canned flowers and storage conditions

**Fig. S1:**
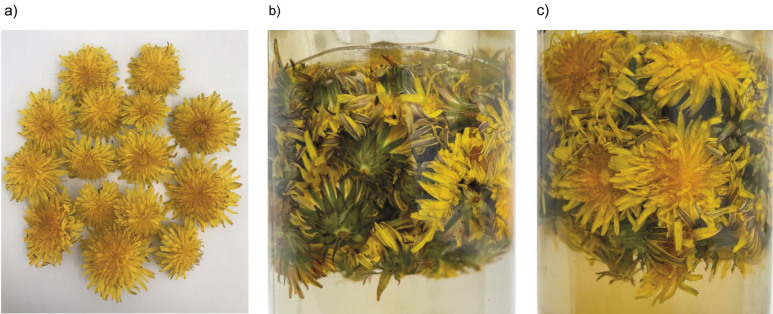
The photographs of: a) fresh dandelion flowers, b) flowers canned in 20 °Bx sucrose syrup, and c) flowers canned in 30 °Bx sucrose syrup

The stems of fresh dandelion flowers were cut off immediately before transportation to the laboratory. Fresh flowers were quickly analysed after being set aside as control samples. All materials used for the canning process were sterilised in an autoclave (MELAG 75+; MELAG, İstanbul, Türkiye) at 121 °C and 1 Pa for 15 min. The canned dandelion flowers were prepared following the methods described in the studies on fruit preserves by Campbell and Padilla-Zakour (*15*) and Christofi *et al.* (*16*). Syrups used as filling media were prepared with sucrose to achieve 20 and 30 °Bx. Flowers weighing 250 g were placed in glass jars, which were then gradually filled with 1.5 L of the syrup, making sure that there were no gaps (Fig. S1). The jars were sealed with lids and pasteurised at 97–98 °C for 20 min. After pasteurisation, the jars were quckly cooled to room temperature under cold running water. The prepared flower preserves were stored in the dark at 25 °C for 30 days. On the 10th, 20th and 30th day, samples of both the flowers and the syrup were collected for analysis of antioxidant activity, TPC and phenolic content. The flower samples taken during storage were first blotted with blotting paper for a few min to absorb any excess syrup before analysis.

### Ultrasound-assisted extraction

The ultrasound-assisted extraction (UP400St ultrasonic processor; Hielscher, Teltow, Germany) was carried out under the following conditions: temperature 40 °C, frequency 40 kHz, power 0.025 W/cm^2^, duration 30 min, *γ*(raw material)=5 g/100 mL and *φ*(EtOH)=64 %, as outlined by Wang *et al.* (*21*).

### 2,2-Diphenyl-1-picrylhydrazyl radical scavenging activity

The free radical scavenging capacity of the samples was determined using a modified version of the 2,2-diphenyl-1-picrylhydrazyl (DPPH) assay described by Brand-Williams *et al.* (*22*). In a 96-well microplate (Nunc™ MicroWell™ 96-well microplates; Thermo Fisher Scientific, Saint-Herblain, France), 20 µL of the diluted sample (0.5 mg/mL) were mixed with 180 µL of a 0.2 mM methanolic DPPH solution (Sigma-Aldrich, Merck, St. Louis, MO, USA). The reaction mixture was incubated (INC 125 F digital Incubator; IKA, İstanbul, Türkiye) at room temperature (15–20 °C) for 25 min, allowing the reaction to proceed. Absorbance was measured at 517 nm using the microplate reader (Thermo Scientific™ Multiskan™ GO microplate spectrophotometer; Thermo Fisher Scientific, Waltham, MA, USA). The absorbance values of the sample (*A*_sample_) and the blank (*A*_blank_, without extract) were then recorded for analysis. DPPH inhibition was calculated as follows:


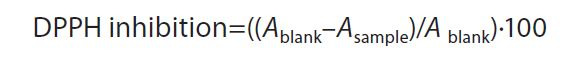
 /1/

### Cupric reducing antioxidant capacity assay

For CUPRAC assay, 500 µL of CuCl_2_ solution (Sigma-Aldrich, Merck) and 500 µL of 1 M glycine solution (Sigma-Aldrich, Merck) (pH=7.0) were transferred into test tubes. Each tube was then supplemented with 500 µL of a neocuproin solution (7.5·10^−3^ M) (Sigma-Aldrich, Merck). After that, 100 µL of a lyophilised extract solution (1 mg/mL) were added, followed by the addition of 550 µL of distilled water. For blank samples, the extract was replaced with distilled water. The mixtures were incubated for 30 min, both at room temperature and in a water bath maintained at 50 °C. The absorbance was measured at 450 nm relative to the blank, using ascorbic acid (Sigma-Aldrich, Merck) as a standard reference (*23*).

### Determination of total phenolic content

The TPC of the samples was assessed spectrophotometrically (Thermo Scientific™ Multiskan™ GO microplate spectrophotometer). For analysis at 760 nm, a mixture was prepared by combining 7.9 mL of distilled water, 0.5 mL of Folin-Ciocalteu reagent (Merck, Darmstadt, Germany) and 1.5 mL of 20 % Na_2_CO_3_ solution (Merck). The resulting solution was incubated at 25 °C for 2 h. Triplicate measurements were carried out using gallic acid (Merck) as the reference standard and the phenolic content was expressed as GAE in mg/g (*24*).

### LC-MS/MS instrumentation and chromatographic conditions

**Fig. 1:**
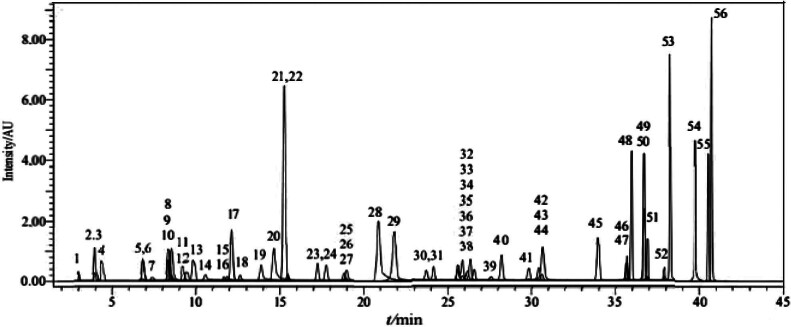
Total ion chromatogram (TIC) of standard phenolic compounds analysed by the LC-MS/MS: 1=quinic acid, 2=fumaric acid, 3=aconitic acid, 4=gallic acid, 5=epigallocatechin, 6=protocatechuic acid, 7=catechin, 8=gentisic acid, 9=chlorogenic acid, 10=protocatechuic aldehyde, 11=tannic acid, 12=epigallocatechin gallate, 13=1,5-dicaffeoylquinic acid, 14=4-hydroxybenzoic acid, 15=epicatechin, 16=vanilic acid, 17=caffeic acid, 18=syringic acid, 19=vanillin, 20=syringic aldehyde, 21=daidzin, 22=epicatechin gallate, 23=piceid, 24=*p*-coumaric acid, 25=ferulic acid-D3, 26=ferulic acid, 27=sinapic acid, 28=coumarin, 29=salicylic acid, 30=cynaroside, 31=miquelianin, 32=rutin, 33=rutin-D3, 34=isoquercitrin, 35=hesperidin, 36=*o*-coumaric acid, 37=genistin, 38=rosmarinic acid, 39=ellagic acid, 40=cosmosiin, 41=quercitrin, 42=astragalin, 43=nicotiflorin, 44=fisetin, 45=daidzein, 46=quercetin-D3, 47=quercetin, 48=naringenin, 49=hesperetin, 50=luteolin, 51=genistein, 52=kaempferol, 53=apigenin, 54=amentoflavone, 55=chrysin, 56=acacetin

Tandem mass spectrometry and Nexera LC-40 XR UHPLC (Shimadzu, Kyoto, Japan) were used to identify phytochemicals present in the samples. Total ion chromatograms (TICs) of standard phenolic compounds are shown in Fig. 1. The reversed-phase UPLC system consisted of LC-30AD model binary pumps, a DGU-20A3R model degasser, a CTO-10ASvp model column oven and a SIL-30AC model autosampler. For chromatographic separation, a reversed phase Agilent Poroshell 120 EC–C18 analytical column with 150 mm length, 2.1 mm inner diameter and 2.7 µm particle size was used. The column temperature was fixed at 40 °C. The gradient elution was prepared using eluent A [(H_2_O with 5 mM NH_4_HCO_2_ (Merck) and 0.1 % HCOOH (Merck)] and eluent B [(MeOH (Sigma-Aldrich, Merck) with 5 mM NH_4_HCO_2_ (Merck) and 0.1 % HCOOH (Merck)]. The following parameters were used in a gradient elution profile: 20 % B (35–45 min), 100 % B (25–35 min) and 20–100 % B (0–25 min).A volume of 5 μL was the injection volume, and 0.5 mL/min was the solvent flow rate. The Shimadzu LCMS-8040 tandem mass spectrometer, which included an electrospray ionization (ESI) source that could function in both positive and negative ionization modes, was used for mass spectrometric detection. LabSolutions software (Shimadzu) was used to gather and analyse LC-MS/MS data. The multiple reaction monitoring, or MRM, approach was used to quantify the phytochemicals. The MRM approach proved to be the most successful in identifying and quantifying the phytochemical compounds according to the tests of various precursor-to-fragment ion transitions. For effective phytochemical fragmentation and maximal transfer of the intended product ions, the collision energies (CE) were tuned. The MS operated with the following parameters: desolvation line temperature of 250 °C, heat block temperature of 400 °C, interface temperature of 350 °C, drying gas (N_2_) flow rate of 15 L/min and nebulizing gas (N_2_) flow rate of 3 L/min (*25*).

### Statistical analysis

Statistical analysis of the obtained data was carried out using Minitab v. 21.3 software (*26*). The results are shown as mean values (S.D.) derived from three independent experiments (*N*≥3). Each sample in the study was replicated at least three times. Variability among the mean results was evaluated using ANOVA and Tukey's multiple comparison test was used for the analysis of variance. Statistical significance was determined at p≤0.05.

## RESULTS AND DISCUSSION

### Phytochemical composition

**Fig. 2:**
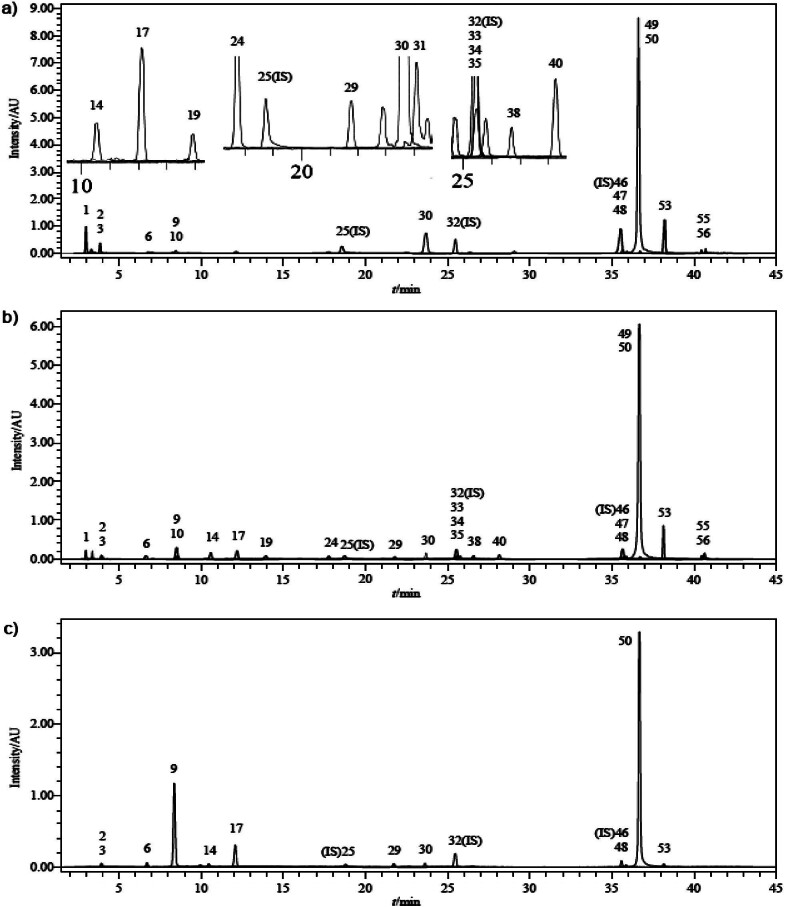
LC*-*MS/MS chromatograms of: a) fresh dandelion flower, b) dandelion flower in 30 ºBx sucrose syrup on day 10 and c) syrup of 30 °Bx on day 20

The therapeutic effects of dandelion flowers are attributed to numerous bioactive compounds (terpenes, flavonoids, phenolics, *etc*.) (*27*). The phytochemical composition of herbal preparations is influenced by factors such as the harvest period, environmental conditions and applied techniques (*28*). The stability of bioactive ingredients, which is critical for the shelf life and bioavailability of the food products to which they are added, is affected by their sensitivity to environmental conditions (oxygen, light, temperature and water) (*29*). In this study, for the first time, comprehensive and sensitive analyses of 56 phytochemicals in both fresh and canned dandelion flowers and their sucrose syrup as filling media were conducted using LC-MS/MS (Table 1). The analysis showed the presence of 24 phytochemicals in different amounts in the fresh flowers, including quercetin, cyranoside (luteolin-7-O-glucoside), cosmosiin (apigenin-7-glucoside), chlorogenic acid, quinic acid, fumaric acid, caffeic acid, luteolin, protocatechuic acid, isoquercitrin (quercetin-3-O-glucoside), *p*-coumaric acid, aconitic acid, protocatechuic aldehyde, apigenin, vanillin, salicylic acid, 4-hydroxybenzoic acid, rutin (quercetin-3-O-rutinoside), rosmarinic acid, hesperetin, naringenin, hesperidin (hesperetin 7-rutinoside), acacetin and chrysin. The polyphenol content in dandelion plants has been reported to be higher in the flowers and leaves ((9.9±0.3) g polyphenols per 100 g dandelion extract) than in the roots ((0.086±0.003) g polyphenols per 100 g dandelion extract) (*30*). Previous studies have identified various flavonoid glycosides in fresh dandelion flowers, such as luteolin, chlorogenic acid, caffeic acid, luteolin-7-diglucoside and luteolin-7-O-glucoside (*31*, *32*), as well as chrysoeriol, monocaffeoyltartaric acid (*33*), ferulic acid, cichoric acid, 3,5-di-O-caffeoylquinic acid, caffeic acid ethyl ester, *p*-hydroxybenzoic acid, 4,5-di-O-caffeoylquinic acid, 3,5-dihydroxybenzoic acid, gallic acid, *p*-coumaric acid, 3,4-dihydroxybenzoic acid and syringic acid (*34*). Furthermore, various flavonoid glycosides, such as quercetin-7-O-glucoside, isorhamnetin-3-O-glucoside, apigenin-7-O-glucoside, and luteolin-7-O-rutinoside, have also been detected (*33*, *35*). When compared with other studies, this study is unique in that it identifies, for the first time, the presence of fumaric acid, quercetin, cosmosiin, isoquercitrin, *p*-coumaric acid, apigenin, 4-hydroxybenzoic acid, protocatechuic acid, aconitic acid, vanillin, salicylic acid, rutin, naringenin, hesperidin, rosmarinic acid, hesperetin, protocatechuic aldehyde, chrysin and acacetin in fresh dandelion flowers. Changes in the content of biologically active components in plants can be influenced by factors including genotype, climate, soil characteristics, vegetative structure, harvest time and various technical practices (*36*). Recent studies have indicated that thermal processing of fruit and vegetables leads to various chemical changes, which may alter the biological activities of phytochemicals (increase, decrease or stability). Therefore, it has been observed that heat-processed foods generally show different biological activities compared to their raw counterparts. The analysis of phenolic compounds showed that 11 of the 56 phenolic compounds were the most dominant in dandelion flowers. These phenolics were ranked in descending order based on their quantities expressed in mg per g of extract as follows: quinic acid 52.3, luteolin 29.5, cyranoside (luteolin-7-O-glucoside) 28.5, chlorogenic acid 22.4, fumaric acid 15.6, caffeic acid 3.98, protocatechuic acid 2.59, quercetin 2.15, cosmosiin 2.07, isoquercitrin 2.00 and apigenin 1.99 (Table 1). The mass fraction of phenolic compounds changed during storage of preserved flowers and syrup due to their different properties. Phytochemicals such as quinic acid, luteolin, luteolin-7-O-glucoside, chlorogenic acid, protocatechuic acid and apigenin were better preserved in flowers stored in 20 and 30 °Bx syrup for 10 and 20 days than for 30 days. These phytochemicals, especially in the 30 °Bx syrup, were found at the highest mass fraction, particularly on day 20. Furthermore, these compounds were also detected in syrup, likely due to their high thermal stability and hydrophilic nature. Analysis indicated lower leakage of phenolic compounds into the syrup on day 20, with this phenomenon being more pronounced in samples preserved in 30 °Bx syrup than those in 20 °Bx syrup. Based on the chromatographic results, it can be concluded that phenolic compounds were best preserved in dandelion flower preserves prepared with 30 °Bx syrup on day 20. On the other hand, phytochemicals such as caffeic acid, quercetin, cosmosiin, isoquercitrin, *p*-coumaric acid, 4-hydroxybenzoic acid, aconitic acid, protocatechuic aldehyde, vanillin, salicylic acid, rutin, hesperidin, rosmarinic acid, hesperetin, naringenin, chrysin and acacetin, despite their hydrophilic properties, were found in very low or undetectable amounts in preserved flowers and syrup due to their moderate or low thermal stability. Fumaric acid, unlike other phytochemicals, has a hydrophobic nature. The amount of this compound consistently decreased during storage in cans with 20 and 30 °Bx syrup, and it is worth mentioning that it did not migrate into the syrup at all. Fig. 2 shows chromatograms of phytochemicals detected in fresh flowers (Fig. 2a), canned dandelion flowers (Fig. 2b) and syrup as filling medium (Fig. 2c). The results of this study are consistent with those of Şengül-Binat and Kırca-Toklucu (*37*), who determined rutin, gallic acid, chlorogenic acid syringic acid and epicatechin concentrations in canned fig samples and filling media during 12 months of storage at 25 °C. They found that the fig juice used in the canning process leaked into the filling medium and resulted in a significant increase in the mass fractions on fresh mass basis of phenolic compounds, namely syringic acid, chlorogenic acid, rutin, epicatechin and gallic acid of 122.64, 22.82, 27.28, 43.32 and 9.96 mg/100 g, respectively.

**Table 1:** Phytochemical composition of fresh dandelion flowers, canned flowers and sucrose syrup as filling medium by LC-MS/MS

				*w*(analyte)/(mg/g)												
No	Analyte	*t*_R_/min	*m*/*z*	A	B	C	D	E	F	G	H	I	J	K	L	M
1	Quinic acid	3.0	190.8	(52.3±1.9)^a^	(38.9±1.4)^a^	(35.44±1.3)^a^	(21.5±0.8)^a^	(46.2±1.7)^a^	(41.7±1.6)^a^	(32.4±1.2)^a^	(19.1±0.7)^b^	(9.3±0.3)^b^	(13.6±0.5)^b^	(5.4±0.2)^b^	(2.23±0.08)^c^	(12.6±0.5)^b^
2	Fumaric acid	3.9	115.2	(15.6±0.1^b^	(9.71±0.08)^b^	(5.66±0.05)^b^	(4.45±0.04)^c^	(12.1±0.1)^b^	(10. 22±0.09)^b^	(7.41±0.06)^b^	N.D.	N.D.	N.D.	N.D.	N.D.	N.D.
3	Aconitic acid	4.0	172.8	(1.61±0.04)^c^	(0.27±0.07)^d^	(0.25±0.06)^d^	(0.10±0.03)^d^	(0.42±0.01)^d^	(0.33±0.08)^d^	(0.20±0.05)^d^	N.D.	N.D.	(0.04±0.01)^d^	N.D.	N.D.	(0.02±0.01)^d^
4	Gallic acid	4.4	168.8	N.D.	N.D.	N.D.	N.D.	N.D.	N.D.	N.D.	N.D.	N.D.	N.D.	N.D.	N.D.	N.D.
5	Epigallocatechin	6.7	304.8	N.D.	N.D.	N.D.	N.D.	N.D.	N.D.	N.D.	N.D.	N.D.	N.D.	N.D.	N.D.	N.D.
6	Protocatechuic acid	6.8	152.8	(2.59±0.09)^c^	(2.04±0.07)^c^	(1.00±0.03)^d^	(0.65±0.02)^d^	(2.42±0.08)^c^	(2.37±0.08)^c^	(1.23±0.04)^c^	(0.22±0.08)^d^	(0.13±0.05)^d^	(0.26±0.09)^d^	(0.15±0.05)^d^	(0.17±0.06)^d^	(0.39±0.01)^d^
7	Catechin	7.4	288.8	N.D.	N.D.	N.D.	N.D.	N.D.	N.D.	N.D.	N.D.	N.D.	N.D.	N.D.	N.D.	N.D.
8	Gentisic acid	8.3	152.8	N.D.	N.D.	N.D.	N.D.	N.D.	N.D.	N.D.	N.D.	N.D.	N.D.	N.D.	N.D.	N.D.
9	Chlorogenic acid	8.4	353.0	(22.4±0.4)^b^	(16.7±0.4)^b^	(13.6±0.3)^b^	(7.3±0.2)^b^	(19.3±0.4)^b^	(18.7±0.4)^b^	(1.3.0±0.3)^b^	(0.64±0.01)^d^	(0.22±0.05)^d^	(0.71±0.01)^d^	(0.36±0.08)^d^	(0.20±0.04)^d^	(0.60±0.01)^d^
10	Protocatechuic aldehyde	8.5	137.2	(1.41±0.05)^c^	(0.56±0.02)^d^	(0.51±0.02)^d^	(0.38±0.05)^d^	(0.75±0.03)^d^	(0.45±0.01)^d^	(0.37±0.01)^d^	N.D.	(0.04±0.02)^d^	(0.06±0.02)^d^	N.D.	N.D.	(0.06±0.02)^d^
11	Tannic acid	9.2	182.8	N.D.	N.D.	N.D.	N.D.	N.D.	N.D.	N.D.	N.D.	N.D.	N.D.	N.D.	N.D.	N.D.
12	Epigallocatechin gallate	9.4	457.0	N.D.	N.D.	N.D.	N.D.	N.D.	N.D.	N.D.	N.D.	N.D.	N.D.	N.D.	N.D.	N.D.
13	Cynarin	9.8	515.0	N.D.	N.D.	N.D.	N.D.	N.D.	N.D.	N.D.	N.D.	N.D.	N.D.	N.D.	N.D.	N.D.
14	4-hydroxybenzoic acid	10.5	137,2	(0.41±0.01)^d^	(0.35±0.08)^d^	N.D.	N.D.	(0.43±0.01)^d^	(0.40±0.01)^d^	(0.42±0.01)d	(0.03±0.01)^d^	N.D.	N.D.	N.D.	N.D.	(0.01±0.01)^d^
15	Epicatechin	11.6	289.0	N.D.	N.D.	N.D.	N.D.	N.D.	N.D.	N.D.	N.D.	N.D.	N.D.	N.D.	N.D.	N.D.
16	Vanilic acid	11.8	166.8	N.D.	N.D.	N.D.	N.D.	N.D.	N.D.	N.D.	N.D.	N.D.	N.D.	N.D.	N.D.	N.D.
17	Caffeic acid	12.1	179.0	(3.98±0.06)^c^	(1.53±0.02)^c^	(1.34±0.02)^c^	(0.71±0.01)^d^	(2.42±0.03)^c^	(1.9±0.03)^c^	(1.04±0.01)^c^	(0.62±0.09)^d^	(0.06±0.01)^d^	(0.47±0.07)^d^	(0.33±0.05)^d^	(0.28±0.04)^d^	(0.57±0.09)^d^
18	Syringic acid	12.6	196.8	N.D.	N.D.	N.D.	N.D.	N.D.	N.D.	N.D.	N.D.	N.D.	N.D.	N.D.	N.D.	N.D.
19	Vanillin	13.9	153.1	(0.12±0.01)^d^	(0.08±0.01)^d^	(0.08±0.01)^d^	(0.08±0.01)^d^	(0.10±0.01)^d^	(0.10±0.01)^d^	(0.11±0.01)^d^	N.D.	N.D.	N.D.	N.D.	N.D.	N.D.
20	Syringic aldehyde	14.6	181.0	N.D.	N.D.	N.D.	N.D.	N.D.	N.D.	N.D.	N.D.	N.D.	N.D.	N.D.	N.D.	N.D.
21	Daidzin	15.2	417.1	N.D.	N.D.	N.D.	N.D.	N.D.	N.D.	N.D.	N.D.	N.D.	N.D.	N.D.	N.D.	N.D.
22	Epicatechin gallate	15.5	441.0	N.D.	N.D.	N.D.	N.D.	N.D.	N.D.	N.D.	N.D.	N.D.	N.D.	N.D.	N.D.	N.D.
23	Piceid	17.2	391.0	N.D.	N.D.	N.D.	N.D.	N.D.	N.D.	N.D.	N.D.	N.D.	N.D.	N.D.	N.D.	N.D.
24	*p*-Coumaric acid	17.8	163.0	(1.83±0.03)^d^	(0.14±0.03)^d^	(0.1±0.02)^d^	(0.09±0.02)^d^	(0.38±0.07)^d^	(0.10±0.02)^d^	(0.11±0.02)^d^	N.D.	N.D.	N.D.	N.D.	N.D.	N.D.
25	Ferulic acid-D3	18.8	196.2	N.A.	N.A.	N.A.	N.A.	N.A.	N.A.	N.A.	N.A.	N.A.	N.A.	N.A.	N.A.	N.A.
26	Ferulic acid	18.8	192.8	N.D.	N.D.	N.D.	N.D.	N.D.	N.D.	N.D.	N.D.	N.D.	N.D.	N.D.	N.D.	N.D.
27	Sinapic acid	18.9	222.8	N.D.	N.D.	N.D.	N.D.	N.D.	N.D.	N.D.	N.D.	N.D.	N.D.	N.D.	N.D.	N.D.
28	Coumarin	20.9	146.9	N.D.	N.D.	N.D.	N.D.	N.D.	N.D.	N.D.	N.D.	N.D.	N.D.	N.D.	N.D.	N.D.
29	Salicylic acid	21.8	137.2	(0.52±0.08)^d^	(0.06±0.01)^d^	(0.06±0.01)^d^	(0.06±0.01)^d^	(0.42±0.07)^d^	(0.27±0.04)^d^	(0.12±0.002)^d^	N.D.	N.D.	N.D.	(0.02±0.01)^d^	(0.03±0.01)^d^	(0.02±0.01)^d^
30	Cyranoside	23.7	447.0	(28.5±1.0)^b^	(21.8±0.8)^b^	(16.8±0.6)^b^	(9.1±0.3)^b^	(23.9±0.9)^b^	(19.5±0.7)^b^	(14.861±0.54)^b^	(4.03±0.1)^c^	(3.07±0.1)^c^	(6.03±0.2)^b^	(2.03±0.07)^c^	(1.02±0.03)^c^	(4.0±0.1)^c^
31	Miquelianin	24.1	477.0	N.D.	N.D.	N.D.	N.D.	N.D.	N.D.	N.D.	N.D.	N.D.	N.D.	N.D.	N.D.	N.D.
32	Rutin-D3-IS	25.5	612.2	N.A.	N.A.	N.A.	N.A.	N.A.	N.A.	N.A.	N.A.	N.A.	N.A.	N.A.	N.A.	N.A.
33	Rutin	25.6	608.9	(0.41±0.01)^d^	(0.08±0.02)^d^	(0.05±0.01)^d^	(0.150.04)^d^	(0.25±0.06)^d^	(0.22±0.05)^d^	(0.20±0.05)^d^	N.D.	N.D.	(0.03±0.01)^d^	N.D.	N.D.	N.D.
34	Isoquercitrin	25.6	463.0	(2.00±0.04)^b^	(0.26±0.06)^d^	(0.2450.05)^d^	(0.152±0.03)^d^	(0.48±0.01)^d^	(0.30±0.07)^d^	(0.13±0.03)^d^	(0.01±0.01)^d^	(0.01±0.01)^d^	(0.11±0.02)^d^	N.D.	N.D.	(0.03±0.01)^d^
35	Hesperidin	25.8	611.2	(0.21±0.07)^d^	(0.07±0.02)^d^	(0.01±0.01)^d^	(0.08±0.03)^d^	(0.06±0.02)^d^	(0.06±0.02)^d^	(0.05±0.02)^d^	(0.01±0.01)^d^	N.D.	(0.01±0.01)^d^	N.D.	N.D.	N.D.
36	*o*-Coumaric acid	26.1	162.8	N.D.	N.D.	N.D.	N.D.	N.D.	N.D.	N.D.	N.D.	N.D.	N.D.	N.D.	N.D.	N.D.
37	Genistin	26.3	431.0	N.D.	N.D.	N.D.	N.D.	N.D.	N.D.	N.D.	N.D.	N.D.	N.D.	N.D.	N.D.	N.D.
38	Rosmarinic acid	26.6	359.0	(0.17±0.02)^d^	(0.13±0.02)^d^	(0.05±0.01)^d^	(0.02±0.01)^d^	(0.12±0.02)^d^	(0.02±0.01)^d^	(0.02±0.01)^d^	N.D.	N.D.	N.D.	N.D.	N.D.	N.D.
39	Ellagic acid	27.6	301.0	N.D.	N.D.	N.D.	N.D.	N.D.	N.D.	N.D.	N.D.	N.D.	N.D.	N.D.	N.D.	N.D.
40	Cosmosiin	28.2	431.0	(2.07±0.01)^c^	(0.09±0.01)^d^	(0.03±0.01)^d^	(0.02±0.01)^d^	(0.10±0.01)^d^	(0.08±0.01)^d^	(0.01±0.01)^d^	N.D.	N.D.	N.D.	N.D.	N.D.	N.D.
41	Quercitrin	29.8	447.0	N.D.	N.D.	N.D.	N.D.	N.D.	N.D.	N.D.	N.D.	N.D.	N.D.	N.D.	N.D.	N.D.
42	Astragalin	30.4	447.0	N.D.	N.D.	N.D.	N.D.	N.D.	N.D.	N.D.	N.D.	N.D.	N.D.	N.D.	N.D.	N.D.
43	Nicotiflorin	30.6	592.9	N.D.	N.D.	N.D.	N.D.	N.D.	N.D.	N.D.	N.D.	N.D.	N.D.	N.D.	N.D.	N.D.
44	Fisetin	30.6	285.0	N.D.	N.D.	N.D.	N.D.	N.D.	N.D.	N.D.	N.D.	N.D.	N.D.	N.D.	N.D.	N.D.
45	Daidzein	34.0	253.0	N.D.	N.D.	N.D.	N.D.	N.D.	N.D.	N.D.	N.D.	N.D.	N.D.	N.D.	N.D.	N.D.
46	Quercetin-D3-IS	35.6	304.0	N.A.	N.A.	N.A.	N.A.	N.A.	N.A.	N.A.	N.A.	N.A.	N.A.	N.A.	N.A.	N.A.
47	Quercetin	35.7	301.0	(2.15±0.03)^c^	(0.61±0.01)^d^	(0.51±0.09)^d^	(0.33±0.06)^d^	(1.90±0.03)c	(1.85±0.03)^c^	(0.83±0.01)^d^	N.D.	N.D.	(0.06±0.01)^d^	N.D.	N.D.	(0.03±0.01)^d^
48	Naringenin	35.9	270.9	(0.44±0.01)^d^	(0.25±0.01)^d^	(0.24±0.01)^d^	(0.24±0.09)^d^	(0.24±0.09)^d^	(0.21±0.08)^d^	(0.211±0.08)^d^	(0.03±0.01)^d^	(0.00±0.01)^d^	(0.05±0.02)^d^	N.D.	N.D.	(0.00±0.01)^d^
49	Hesperetin	36.7	301.0	(0.12±0.04)^d^	(0.05±0.02)^d^	(0.02±0.01)^d^	(0.03±0.01)^d^	(0.105±0.03)^d^	(0.05±0.02)^d^	(0.05±0.02)^d^	N.D.	N.D.	N.D.	N.D.	N.D.	N.D.
50	Luteolin	36.7	284.8	(29.5±0.9)^b^	(21.5±0.7)^b^	(19.9±0.7)^b^	(12.94±0.40)^b^	(24.3±0.8)^b^	(22.4±0.7)^b^	(16.6±0.5)^b^	(1.16±0.03)^c^	(0.51±0.06)^d^	(2.00±0.06)^c^	(1.01±0.03)^c^	(0.76±0.02)^d^	(1.34±0.04)^c^
51	Genistein	36.9	269.0	N.D.	N.D.	N.D.	N.D.	N.D.	N.D.	N.D.	N.D.	N.D.	N.D.	N.D.	N.D.	N.D.
52	Kaempferol	37.9	285.0	N.D.	N.D.	N.D.	N.D.	N.D.	N.D.	N.D.	N.D.	N.D.	N.D.	N.D.	N.D.	N.D.
53	Apigenin	38.2	268.8	(1.99±0.03)^c^	(1.85±0.03)^c^	(1.47±0.02)^c^	(1.54±0.02)^c^	(1.64±0.02)^c^	(1.44±0.02)^c^	(1.26±0.02)^c^	(0.19±0.03)^d^	(0.00±0.01)^d^	(0.22±0.04)^d^	(0.07±0.01)^d^	(0.04±0.01)^d^	(0.07±0.01)^d^
54	Amentoflavone	39.7	537.0	N.D.	N.D.	N.D.	N.D.	N.D.	N.D.	N.D.	N.D.	N.D.	N.D.	N.D.	N.D.	N.D.
55	Chrysin	40.5	252.8	(0.10±0.03)^b^	(0.09±0.03)^d^	(0.08±0.03)^d^	(0. 10±0.03)^d^	(0.07±0.02)^d^	(0.01±0.01)^d^	N.D.	N.D.	N.D.	N.D.	N.D.	N.D.	N.D.
56	Acacetin	40.7	283.0	(0.08±0.03)^d^	(0.11±0.04)^d^	(0.10±0.03)^d^	(0.04±0.01)^d^	(0.02±0.01)^d^	(0.01±0.01)^d^	(0.01±0.01)^d^	N.D.	N.D.	N.D.	N.D.	N.D.	N.D.

N.D.=not detected, N.A.=not applicable. Samples: A=fresh dandelion flower, B, C and D=dandelion flower in 20 °Bx syrup on day 10, 20, and 30, E, F and G=dandelion flower in 30 °Bx syrup on day 10, 20 and 30, H, I and J=20 °Bx syrup on day 10, 20 and 30, K, L and M=30 °Bx syrup on day 10, 20 and 30. Results are expressed as mean value±S.D., *N*=3 and the values were calculated according to negative control. Values with different letters in superscript in the same row are significantly different (p<0.05).

### Antioxidant activity and total phenolic content

The DPPH test measures the ability of antioxidants in herbal preparations to neutralise free radicals, while the CUPRAC test evaluates their ability to reduce Cu^2+^ ions to Cu^+^ ions (expressed as Trolox equivalents). In both DPPH and CUPRAC analyses, the highest antioxidant activity was found in sample A (89.6 % and 0.8 mmol/g, respectively) (Table 2). The results closest to this value were observed in samples E (86.3 % and 0.7 mmol/g), F (78.1 % and 0.7 mmol/g), B (74.9 % and 0.6 mmol/g), and C (72.9 % and 0.6 mmol/g), respectively. DPPH and CUPRAC activities were significantly lower in the syrup than in the fresh and preserved flowers (p<0.05). The lowest DPPH (56.6 %) and CUPRAC (0.5 mmol/g) content was found in sample I, which was canned flowers in a 20 °Bx syrup. The lowest antioxidant activity measured by DPPH (41.5 %) and CUPRAC (0.3 mmol/g) was found in 30 °Bx syrup (p<0.05). A proportional relationship was observed between the TPC and the antioxidant activity tests. The highest TPC, expressed as GAE, in sample A was 367.4 mg/g extract (p<0.05). During storage, these values were found to be lower in canned dandelion in both 20 and 30 °Bx syrups (Table 2). The TPC in syrup was also lower than in the fresh and canned flowers. In particular, the lowest TPC was observed in sample I (20 °Bx, 205.4 mg/g) and sample L (30 °Bx, 171.8 mg/g extract) (p<0.05). From these results, it can be concluded that the antioxidant activity of canned flowers was maintained in 20 and 30 °Bx sucrose syrups compared to fresh flowers for 10 and 20 days, respectively, but decreased by day 30. The results also show that phenolic compounds with hydrophilic properties, which leach into the syrup, are present in different amounts. It is noteworthy that a lower transfer of phenolic compounds from canned flowers prepared with 30 °Bx syrup into the filling medium was observed than for those prepared with 20 °Bx. In fact, TPC was found to be lowest in the samples in the 30 °Bx filling medium on day 20 (p<0.05). These data indicate that at 30 °Bx, the phenolic compounds of canned flowers are better preserved and fewer are transferred into the syrup. Dedić *et al*. (*38*) prepared aqueous ethanol extracts of dandelion using many extraction methods, such as maceration at ambient and increased temperature, ultrasonic extraction, and soxhlet extraction. They then examined the root, leaf, stem and floral components of the plant. The authors stated that the concentration of phenolic compounds was higher in the floral and foliar parts of the plant than in the root, with the maximum antioxidant activity detected in the aqueous ethanol extract obtained in Soxhlet extraction. Nowak *et al*. (*39*) extracted fresh and dried dandelion leaves, flowers and roots in an ultrasonic bath with ethanol volume fractions of 40, 70 and 96 %, and extraction periods of 15, 30 and 60 min. It was observed that raw material, solvent and extraction time affected the antioxidant activity of dandelions. Dried flower extracted for 30 min with 70 % ethanol had the highest DPPH activity, while dried leaf extracted with 40 % ethanol had the highest FRAP reduction capability. Ivanov (*27*) reported that total phenolics, chicoric acid content, and antioxidant activity (DPPH, FRAP and CUPRAC) were increased in 50 % ethanol extracts of dandelion leaves. Miłek and Legath (*40*) extracted phenolic compounds from dandelion flowers and leaves by ultrasonic extraction with solvents (methanol, ethanol and acetone) at a volume fraction of 70 %. The maximum extraction of phenolics from leaves was achieved using acetone, followed by methanol and ethanol. The total phenolic content of the extracts of *Taraxacum officinale* was determined to be (362.14±6.76) µM. The parts of the plant used, the climatic conditions of the region where the plant is collected, the type, duration and temperature of extraction, the polarity of the solvent, the solubility of polyphenols, and their interactions with other compounds significantly affect antioxidant activity. Additionally, the lipophilic/hydrophilic properties of plant compounds should be considered. Moreover, thermal processing conditions can accelerate oxidation and other degenerative reactions, leading to the loss of natural antioxidants. Considering these factors, the results of this study can differ from those of other studies. Several studies have investigated the antioxidant effects of fresh dandelion flowers in both *in vitro* and *in vivo* media. In a study consistent with our findings, it was reported that methanolic extract of dandelion flower had an inhibition rate of 95 % (*41*). Antioxidant compounds in dandelion flowers have been shown to inhibit DNA and liposome oxidation induced by peroxyl and hydroxyl radicals *in vitro* (*42*). Another study found that dandelion flowers were more effective than leaves in inhibiting plasma protein and lipid oxidation *in vitro* (*9*). The authors indicated that the reducing activity of dandelion flowers is equivalent to 40 % of ascorbic acid and the inhibitory activity of fresh flower extracts against damage induced by reactive oxygen species and nitric oxide could be related to caffeic acid, chlorogenic acid, luteolin and luteolin-7-O-glucoside. Dandelion polyphenols have been shown to reduce the production of nitric oxide, prostaglandin E2, TNF-α and IL-1 in lipopolysaccharide-stimulated RAW264.7 cells. Additionally, luteolin and luteolin-7-O-glucoside from dandelion flower extracts have been shown to reduce the expression of both inducible nitric oxide synthase and cyclooxygenase 2 (*43*). According to a study by Burda and Oleszek (*44*), the hydroxyl radical-suppressing effect of dandelion flower extract could be partly due to the presence of phenolic components such as flavonoids and coumaric acid. Furthermore, the DPPH radical scavenging activity of dandelion flower extract has been associated with the presence of luteolin-7-glucoside. Hassan *et al.* (*45*) observed that long-term use of dandelion flower extract (300 mg/kg body mass per day) in rats played a crucial role in combating oxidative stress. The study concluded that the flavonoids, phenolic acids and terpenoids found in fresh dandelion flowers, together with other antioxidants, could protect the human body against the pathological effects of free radicals (*31*, *32*). Therefore, it is suggested that the natural compounds found in dandelion have antioxidant, anticoagulant and anti-clotting activity, making them potentially useful in the prevention and treatment of commonly occurring cardiovascular diseases. Many studies have indicated that canned foods contain similar amounts of certain nutrients to fresh or frozen foods. For example, more than 30 % of phenolic compounds in canned peach and apricot varieties diffused into the syrup (*15*). In addition, it has been reported that canned fruit and syrups have higher phenolic content after 6 months of storage at 20 °C. Researchers have suggested that syrup consumption or secondary use may be important to increase total phenolic intake from canned fruit. In the study by Chaovanalikit and Wrolstad (*46*), approx. 50 % of the phenolic compounds in canned cherries were found to pass into the syrup. Asami *et al.* (*47*) observed that different storage periods may alter the quantity of phenolic compounds. In the study by Şengül-Binat and Kırca-Toklucu (*37*), TPC in the filling media of canned figs was analysed during canning and storage. They observed an increase in the TPC of figs canned in juice and syrup immediately after canning, while a decrease in TPC was observed in the canned fig juice itself. In addition, 6 and 12 months of storage resulted in a 25–35 % reduction in the TPC of canned figs. These results indicate that, although storage leads to a gradual decline, the canning process effectively preserves a considerable portion of the phenolic compounds and antioxidant capacity of figs. However, antioxidant compounds can be oxidised and degraded due to thermal processing. Various factors, such as heating temperature, duration and type, can influence the stability of these compounds. Phenolic compounds, being water-soluble, may leach into their surroundings, particularly in fruit immersed in syrup or filling medium (*17*). Thermal treatment can significantly affect the absorption of phenolic compounds by the body, resulting in a notable reduction in the chemical composition of foods, particularly phenolic compounds (*48*). This process is often linked to a substantial decrease in the antioxidant activity. Additionally, the storage itself can contribute to a decrease in the TPC of food products (*49*). The cooking of plant products may break down cell wall components and cause the release of molecules or leaching of water-soluble polyphenols into the surrounding environment. Polyphenols can also degrade at increased temperatures (*50*). In contrast to our findings, Wang *et al.* (*51*) reported a significant increase in the antioxidant activity of canned lychee pulp following heat treatment at 121 °C. Similarly, Chen *et al.* (*52*) showed that high-pressure treatment and thermal processing (121 °C, 3 min) increased bioactive compounds and total antioxidant activity in green asparagus juice. Yahya *et al.* (*53*) determined the TPC, expressed as GAE, of canned fruit to be 95.16 mg/100 g in pineapple, 47.69 mg/100 g in longan, 51.80 mg/100 g in litchi, and 27.53 mg/100 g in rambutan. In particular, fruit in the form of syrup formhad higher TPC than canned fruit. The radical scavenging capacity, expressed as Trolox equivalent, of canned pineapple (41.79 μmol/100 g), rambutan (39.35 μmol/100 g), longan (41.67 μmol/100 g) and lychee (39.76 μmol/100 g) were determined using the DPPH assay. Interestingly, syrup samples had higher radical scavenging activity than canned fruit. Durst and Weaver (*54*) reported that canned peaches had 1.5 times higher antioxidant activity than fresh peaches, with no significant decrease observed after 3 months of storage.

**Table 2:** Evaluation of DPPH, CUPRAC and TPC of fresh and canned dandelion flowers and sucrose syrup as filling media

Sample	DPPH inhibition/%	CUPRAC as *b*(TE)/(mmol/g)	TPC as *w*(GAE)/(mg/g)
A	(89.6±0.66)^a^	(0.8±0.2)^a^	(367.4±0.8)^a^
B	(74.9±0.5)^b^	(0.6±0.2)^c^	(321.6±0.7)^b^
C	(72.9±0.5)^b^	(0.6±0.1)^c^	(289.1±0.6)^c^
D	(63.5±0.4)^c^	(0.57±0.09)^c^	(248.1±0.5)^d^
E	(86.3±0.5)^a^	(0.7±0.2)^b^	(353.6±0.8)^a^
F	(78.1±0.5)^b^	(0.7±0.2)^b^	(320.9±0.6)^b^
G	(69.3±0.4)^c^	(0.6±0.1)^c^	(302.0±0.6)^c^
H	(64.0±0.4)^c^	(0.6±0.1)^d^	(281.8±0.5)^c^
I	(56.6±0.3)^d^	(0.48±0.08)^e^	(205.4±0.4)^d^
J	(60.1±0.3)^d^	(0.6±0.1)^d^	(215.6±0.4)^d^
K	(48.2±0.2)^e^	(0.4±0.1)^e^	(194.8±0.3)^e^
L	(41.5±0.2)^f^	(0.3±0.1)^f^	(171.8±0.2)^f^
M	(58.7±0.3)^d^	(0.56±0.07)^c^	(232.3±0.4)^d^

DPPH=2,2-diphenyl-1-picrylhydrazyl, CUPRAC=cupric reducing antioxidant capacity, TPC=total phenolic content, TE=Trolox equivalent, GAE=gallic acid equivalent. Results are mean value±S.D. (*N*=3). Different letters in superscript within the same column show significant differences (p<0.05). Samples: A=fresh dandelion flower, B, C and D=dandelion flower in 20 °Bx syrup on day 10, 20 and 30. E, F and G=dandelion flower in 30 °Bx syrup on day 10, 20 and 30, H, I and J=20 °Bx syrup on day 10, 20 and 30, K, L and M=30 °Bx syrup on day 10, 20 and 30

## CONCLUSIONS

The study demonstrates an innovative approach to extend the short shelf life of dandelion flowers by canning while preserving their valuable phytochemical content and compounds with antioxidant properties. This method offers a practical solution to extend the shelf life of dandelion flowers beyond their natural availability and make their health benefits accessible all year round. In particular, the optimal conditions for canned flowers were found to be 30 °Bx sucrose syrup for 20 days, providing important insights for producers and consumers. The results emphasise the superior preservation ability of sucrose syrup, even though a decrease in antioxidant activity is observed after 10 days. An important innovation is the recommendation to consume the flowers together with the filling medium to maximise the nutritional benefits. In addition, the study introduces the idea of exploring alternative filling media, such as fruit juices, which could open up new ways to improve both the nutritional value and consumer appeal of preserved dandelion flowers. This research not only provides practical guidelines for manufacturers, but also contributes to the growing body of knowledge on the preservation of natural products rich in phytochemicals, and emphasises its novelty and importance in the field of food science and nutrition.
